# The Effect of Scanning Strategy on Intraoral Scanner’s Accuracy

**DOI:** 10.3390/dj10070123

**Published:** 2022-07-04

**Authors:** Nikolaos A. Gavounelis, Chrysoula-Maria C. Gogola, Demetrios J. Halazonetis

**Affiliations:** School of Dentistry, National and Kapodistrian University of Athens, 11527 Athens, Greece; marichrysa.gogola@gmail.com (C.-M.C.G.); dhalaz@dent.uoa.gr (D.J.H.)

**Keywords:** intraoral scanner, 3D diagnosis and treatment planning, scan strategy, image stitching, accuracy

## Abstract

The purpose of the present study was to examine the impact of scanning strategy on trueness and precision of the impression acquired from an intraoral scanner. Fifteen complete-arch, mandibular, post-orthodontic treatment casts were scanned with a laboratory scanner (Identica SE 3D, Medit) as the gold standard, and with an intraoral scanner (i500 Medit) following three different paths of the scanning head over the arch (scanning strategies A, B, and C). The hand scans were performed twice by one examiner and repeated by a second examiner, resulting in 180 triangular mesh surfaces (digital casts). The meshes were superimposed on the gold standards using the Viewbox 4 software. The closest distances between the meshes were computed and trueness and precision were evaluated using a General Linear Model. An interaction was found among the examiner and strategy. The accuracy of complete-arch impressions was affected by the scanning strategy; the manufacturer’s recommended strategy (A) was statistically significantly better (*p* < 0.05) than B and C, which were similar. An average accuracy below 50 μm, which is clinically acceptable in most orthodontic procedures, was achieved with all the examined scanning strategies.

## 1. Introduction

Several systematic reviews and meta-analyses on digital scanning have been published, evaluating accuracy, patient preference, and time reduction [1,2,3]. Specifically in orthodontics, where fully dentate scans are usually acquired, digital scanning simplifies the diagnostic and treatment procedures, results in a better time management [3,4] and highly satisfied patients [4,5,6], achieves a high accuracy [7,8,9], and requires a smaller learning curve [10,11].

For correct scanning, the clinician positions the scanned object at the center of the viewfinder [11] and moves the head of the intra-oral scanner (IOS) along a specific path, the so-called scanning strategy. The impact of the scanning strategy on the accuracy of the impression is not yet fully determined. Scanning strategies different than those suggested by the IOS manufacturer may lead to a significantly lower accuracy [12,13]. Anh et al. [14] demonstrated that the precision of the digital model depends on the starting point of the scan. Oh et al. [15] concluded that the vertical rotation of the IOS should be avoided. Medina et al. [16] and Passos et al. [17] tested multiple IOS systems and found that scan strategy affects the accuracy of IOSs differently, depending on their data capturing method. In maxillary edentulous cases, the optimum scanning strategy may depend on the surface characteristics and the presence of palatal rugae [18]. IOS systems that use active triangulation technology have not been adequately tested.

All scanning systems create the three-dimensional (3D) model by merging (‘stitching’) several images taken under different viewpoints [19]. The scan strategy is closely related to the image stitching software; if scanner movement is too fast or has extreme changes in orientation, the stitching process may be compromised [20,21].

The aim of this study was to examine the effect of scanning strategy on trueness and precision of the digital impression. The manufacturer’s recommended strategy was compared with two alternatives: one without extreme changes in the IOS head direction and one with continuous changes. The null hypothesis was that scanning strategies do not affect the accuracy of the impressions.

## 2. Materials and Methods

### 2.1. Sample

A sample size estimation was performed in advance of the study, based on an ANOVA repeated measures, within subjects test. Using an alpha level of 0.05, a power of 0.8, an effect size of 0.4, and an estimated correlation between measurements of 0.5, we arrived at a sample size of 12 (G*Power 3.1.9.7, Universität Kiel, Kiel, Germany). This was augmented to 15 to compensate for potential overestimation of the correlation.

Thus, our sample comprised 15 complete-arch mandibular post-treatment permanent dentition dental casts from patients of the Department of Orthodontics, School of Dentistry, National and Kapodistrian University of Athens (Athens, Greece). Informed consent was obtained from all participants and/or their legal guardians.

### 2.2. Scanning Procedure

The casts were scanned with the laboratory scanner Identica SE 3D (Medit, Seoul, Korea), which was considered the gold standard, and with the IOS i500 (version 1.1.1, Medit, Seoul, Korea) following three different scanning strategies (see below). The scans, following all strategies, were performed twice by examiner 1 and were repeated twice by examiner 2, totaling 180 meshes. Examiner 1, during the first session, was aiming for a detailed scan without time limit; a time limit of 60 secs (recommended scanning time by Medit) was set for the remaining scans. Additionally, 5 casts were scanned twice with the laboratory scanner in order to validate its precision. Here we follow the ISO 5725-1 definitions of trueness, precision, and accuracy [22].

The Medit Link software (version 2.2) was used for intraoral scanning. The settings were set to ‘mandible scanning’, 16 mm scanning depth and filtering level of 1. The high-resolution option was deactivated. The IOS was calibrated before each scan session.

**Figure 1:**
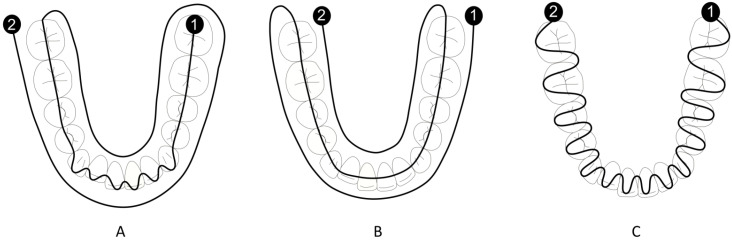
(**A**) Scanning strategy A. (**B**) Scanning strategy B. (**C**) Scanning strategy C. The scan starts at point 1 and proceeds with a continuous movement to point 2.

In scanning strategy A, recommended by the manufacturer, the scan was continuous, starting from the occlusal surface of the lower left posterior teeth, followed by the anterior teeth with an alternating labio-lingual movement, and finally the occlusal surface of the lower right posterior teeth. The scan was completed with the impression of the lingual and labial surfaces (Figure 1A).

In scanning strategy B, the scan was performed in a single uninterrupted motion. It started from the buccal surface with a left-to-right direction, then captured the occlusal surfaces and finally the lingual (Figure 1B).

In scanning strategy C, the scan was performed in a continuous labial-lingual motion, with a left-to-right direction (Figure 1C).

At the end of each scan, scanning time was recorded and the files were post-processed by Medit Link. The options of not filling holes and excluding unreliable data were selected and then files were exported in object file (obj) format.

### 2.3. Mesh Superimposition

To compare the impressions acquired with IOS to the reference casts from the laboratory scanner, we used the Viewbox 4 software (version 4.1.0.10 BETA, dHAL software, Kifissia, Greece). The meshes were imported without further processing. Each mesh acquired from the Medit Identica consisted of approximately 1,000,000 vertices and 2,000,000 triangles. The corresponding meshes from the Medit i500 consisted of around 140,000 vertices and 280,000 triangles. Each impression acquired from the laboratory scanner (reference impression) was superimposed on the corresponding one from the IOS i500, using the crowns of the teeth only, and not the whole cast, and were performed through the iterative closest point algorithm (ICP). The distances between the closest pair of points of the superimposed meshes were computed (around 75,000 distances per cast). The same procedure was followed for each mesh acquired twice from the laboratory scanner.

### 2.4. Statistical Analysis

SPSS statistics (version 26.0 IBM Corp. Armonk, NY, USA) was used for the statistical analysis. A General Linear Model was created for the data acquired from the superimpositions between meshes from IOS and the laboratory scanner. The dependent variable was the mean value of absolute distances between casts and the fixed factors were examiner, strategy, and repeat. Post-hoc analysis (Tukey, Scheffe) was conducted to assess which strategy was statistically significantly different. The level of significance was set at 0.05.

For the precision study, the mean value of the distances between the closest pair of points of each superimposed mesh was computed. This was conducted for examiner 1 and examiner 2, separately. Hence, there were two means for every cast scanned with the same strategy and their standard deviation (SD) was estimated. This process was repeated for every cast. The mean value of the SDs represents the precision of the IOS for the examined scanning strategy.

## 3. Results

The results regarding the precision of the laboratory scanner are shown in Table 1.

**Figure 2:**
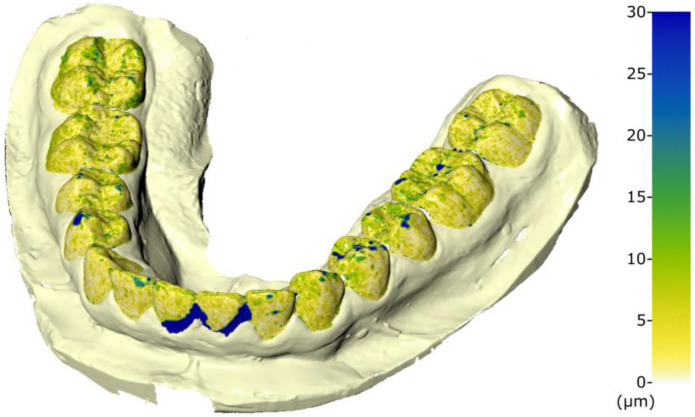
Superimposition of 2 impressions of the same cast, both acquired with the laboratory scanner. The color-map indicates its precision. Values in μm.

Figure 2 shows the superimposition between two meshes scanned with Identica SE 3D (Medit).

The mean trueness values of the IOS were 37.5 (±12.5) μm, 44.8 (±17.3) μm, and 43.9 (±20.0) μm, for scanning strategies A, B, and C, respectively (Table 2).

**Figure 3:**
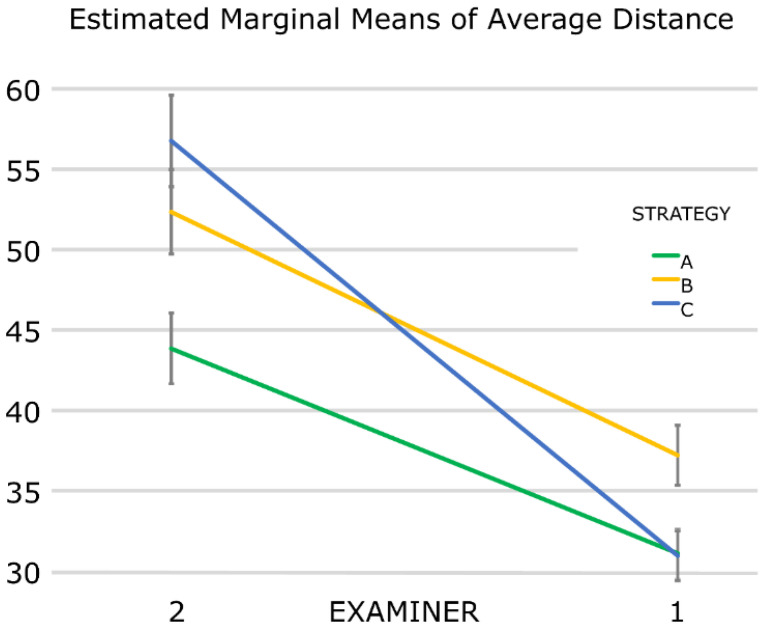
Comparison of the achieved trueness between the examiners for each scanning strategy. Error bars: 95% confidence interval. Values in μm.

Examiner one got better results than examiner two on all strategies. However, there was an interaction between examiner and strategy, since examiner one was equally better in strategies A and B but much better in strategy C than examiner two, who had the worst performance using this strategy (higher error). This fact implies that strategy C might be examiner-sensitive (Figure 3).

Both examiners achieved less accurate results during the second scan session. Additionally, the second scan session results were similarly less accurate for all scanning strategies. No interaction between the examiner and the repeat or between the strategy and the repeat was detected. Strategy A was the most accurate in both sessions.

The General Linear Model demonstrated that the mean value of the absolute distances depends on three factors: the examiner, the strategy, and the repeat. In addition, there was a positive correlation between the examiner and the strategy (Table 3 and Table 4).

**Figure 4:**
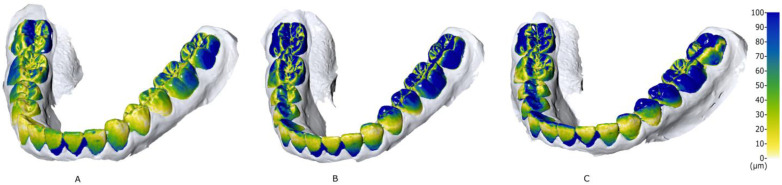
Representative superimpositions for each scanning strategy (**A**–**C**). The color-map indicates the trueness of Medit i500. Premolars and molars of the lower left quadrant presented inferior trueness, probably because this region was the scan’s starting point for all strategies. Values in μm.

Post-hoc analysis demonstrated that strategy A was statistically significantly better and differed from B and C, which were similar (Figure 4).

The mean precision values of the IOS were 2.7 (±2.4) μm, 4.7 (±3.0) μm, and 3.4 (±2.7) μm for scanning strategies A, B, and C, respectively (Table 5).

Scanning strategy C was faster than A and B (Table 6). The 1st scan session lasted 69 s while the 2nd was 47 s, on average.

## 4. Discussion

The null hypothesis was rejected, since scanning strategy A provided statistically significantly better results, albeit with little clinical consequence. Strategy C was 8 s faster, but this is also clinically negligible.

The in vitro design of this study allowed for a high number of scans (*n* = 180) and ensured similar scanning conditions. In addition, a high precision reference scan from a laboratory scanner was available for testing trueness. However, dental casts do not simulate the clinical situation adequately; saliva, blood, a patient’s movement, a limited work field, moving soft tissues, pharyngeal reflexes, the translucency of the oral mucosa, lighting conditions, malaligned or crowded arches, and metal appliances with reflective surfaces are factors not taken into account [14,15,23,24,25,26,27]. Trueness and precision are significantly affected by the reflection, refractive index, and translucency of the substrate [28,29].

In the current study all scans were performed by two examiners, to investigate the interaction between the examiner and the scanning strategy. Examiner one achieved better accuracy results on all strategies, probably because of more experience than examiner two [30,31].

This study is one of the few to investigate the effect of scan strategy on the accuracy of active triangulation IOS. In active triangulation, the object’s distance is computed from the image coordinates of two different points of view [20]. Active triangulation scanners are affected by different substrates more than those using confocal microscopy [28]. Medina et al. [16] investigated the impact of scanning strategy on the accuracy of four IOS systems, two using confocal microscopy, one active wavefront sampling, and one active triangulation. Only a confocal IOS was depended on the scanning strategy. It attained better accuracy results when a sequential strategy (similar to the strategy C of the current study) was followed. In contrast, the active triangulation IOS that we examined was affected by scanning strategy, with the sequential strategy leading to inferior results. This could be attributed to the different IOS system used, regarding both hardware and software.

To create the 3D model, all scanning systems use “image stitching” algorithms, which may produce inaccuracies, especially if the surfaces to be joined together do not have salient features. In scanning strategy A, which was statistically significantly better, the scan started from the occlusal surfaces of the posterior teeth, an area with complex morphology. In scanning strategies B and C, the scan started from the buccal surface of the posterior teeth, a region with simpler morphology, potentially leading to errors in the image stitching process. A previous study [14] also indicated a relation between the starting point and the accuracy of the IOS, but the starting points did not include different tooth surfaces and the scanners were based on the principle of confocal microscopy. In contrast, Oh et al. [15], using the same IOS and software as with the current paper, but different scanning strategies, noted that the starting position of the scan does not affect accuracy.

In this study we focused on complete dentate arches, as frequently encountered in orthodontics. In patients whose dentitions are malaligned or crowded, who have orthodontic appliances, such as brackets with deep undercuts and highly reflective or translucent surfaces, scanning might not be as accurate [2,14,27]. In edentulous cases, accuracy may depend significantly on the topography of the mucosa and the presence of characteristic structures such as the palatal rugae; in such cases, scanning strategy seems to be an important factor [18].

The accuracy of the IOS regarding the individual axes (x, y, z) has not been fully determined [32]. A recent study [15] emphasized that rotations and vertical movements of the scanner head should be minimized, since a change of direction may disrupt the image-stitching process. We observed significantly lower accuracy in strategy C, in which rotations dominate. Interestingly, strategy B, in which the IOS was held mostly horizontally, led to a similar inferior accuracy. This is in agreement with the outcomes of Passos et al. [17], who observed that the sequential strategy led to significantly inferior results than the mainly linear, dominant strategy. However, the results of the sequential strategy were similar with many other linear strategies.

In the current study, the i500 (Medit) reached errors below 50 μm using any of the three scanning strategies, similar to previous studies [33,34]. This error is clinically acceptable for orthodontic diagnosis and treatment planning [26,35,36,37]. Intra-arch linear measurements, such as intercanine width and Bolton analysis, can be reliably achieved [35,38]. However, other orthodontic procedures may need a higher accuracy. Errors of 50 μm may be significant, compared to commonly planned interproximal enamel reductions (IPR) of 100–500 μm per tooth [39]. Furthermore, the recording of the occlusal contacts could be inaccurate [40,41] as 50–100 μm are considered as a contact [42], with the traditional articulating paper having a thickness of 80 μm. However, bite registration is a more complex procedure, not examined in this study.

The accuracy of 50 μm is clinically acceptable for 3D printing and the fabrication of orthodontic appliances. Digital workflow can manufacture single unit fixed dental prostheses within the 120 μm maximum marginal misfit [43]. Accordingly, it would be reasonable to assume that digital workflow may also be used for the fabrication of orthodontic bands, and even more so for the 3D printing of dental casts for diagnosis and manufacturing of orthodontic appliances [44,45,46], and for direct 3D printing of retainers [47] and indirect bonding transfer trays [48].

Based on these results, the i500 (Medit) can acquire clinically acceptable scans, using any of the three strategies. However, because inaccuracies in scanning can lead to the accumulation of errors in the following steps of the digital workflow, the manufacturer’s recommended strategy may be preferable. Scanning strategies that include rotation of the IOS head might be examiner-sensitive. Scanning the anterior region of the arch proved to be challenging on several casts. Superimpositions of all scanning strategies (Figure 4) confirm previous results [11,14] that labial inclination of the anterior teeth and the resulting shadowing from the occlusal view lead to an inferior scanning accuracy, mostly at interproximal surfaces. Such circumstances can occur in patients with severe crowding, possibly leading to errors in appliance manufacturing [14]. In all cases, meticulous scanning without a time limit is suggested.

Future in vivo studies should include IOSs based on all image acquisition principles to further understand the interaction between scanning strategy and scanner technology. Future research may concentrate on the IOS software, especially the image-stitching algorithm and guided scanning strategies, which could lead to improvements in accuracy.

## 5. Conclusions

The i500 (Medit) produced digital complete arch impressions with an average trueness value below 50 μm and an average precision value below 5 μm, using any of the examined scanning strategies.

The manufacturer’s recommended scanning strategy was statistically more accurate, but the observed difference of 6–7 μm between the recommended and the alternative strategies is clinically negligible.

## Figures and Tables

**Table 1:** Descriptive statistics for evaluating the precision of the laboratory scanner.

Mesh	Mean (±SD)	Median
1	5.1 (±16.8)	2.8
2	10.0 (±31.4)	4.7
3	6.6 (±33.5)	3.3
4	7.5 (±28.6)	3.7
5	9.6 (±37.7)	4.0

Values in μm.

**Table 2:** Results of the trueness study of the IOS. Mean and standard deviation for each scanning strategy, examiner, and session.

	Strategy A		Strategy B		Strategy C	
	**Examiner 1**	**Examiner 2**	**Examiner 1**	**Examiner 2**	**Examiner 1**	**Examiner 2**
Session 1	26.8 (±6.5)	39.6 (±14.3)	29.3 (±6.1)	47.2 (±15.7)	27.4 (±8.6)	48.4 (±17.5)
Session 2	35.5 (±7.6)	48.1 (±10.2)	45.1 (±12.1)	57.5 (±19.8)	34.5 (±12.5)	65.1 (±16.3)
Overall	37.5 (±12.5)		44.8 (±17.3)		43.9 (±20.0)	

Values in μm.

**Table 3:** Results parameter estimates of the General Linear Model.

Parameter	Coefficient Estimate	95% Confidence Interval	*p*-Value
Intercept	36.5	31.5 to 41.6	0.000
Examiner 2	25.8	19.2 to 32.4	0.000
Strategy A	0.1	−6.5 to 6.8	0.965
Strategy B	6.3	−0.4 to 12.9	0.063
Session 1	−11.2	−15.0 to −7.4	0.000
Examiner 2 × strategy A	−13.0	−22.4 to −3.7	0.006
Examiner 2 × strategy B	−10.7	−20.0 to −1.3	0.026

Reference levels: Examiner = 1 Strategy = C, Session = 2.

**Table 4:** Results tests of between-subjects effects of General Linear Model.

Source	Type III Sum of Squares	df	Mean Square	F	*p*-Value
Corrected Model	23,355.3	6	3892.6	23.2	0.000
Intercept	318,109.7	1	318,109.7	1893.3	0.000
Examiner	14,398.6	1	14,398.6	85.7	0.000
Strategy	1896.7	2	948.4	5.6	0.004
Session	5611.2	1	5611.2	33.4	0.000
Examiner × strategy	1448.8	2	724.4	4.3	0.015

R Squared = 0.446 (Adjusted R Squared = 0.426). Values in μm.

**Table 5:** Results of the precision study of the IOS. Mean of the standard deviations (±standard deviation) for each scanning strategy and examiner.

	Strategy A		Strategy B		Strategy C	
	**Examiner 1**	**Examiner 2**	**Examiner 1**	**Examiner 2**	**Examiner 1**	**Examiner 2**
Mean of SDs	2.9 (±2.5)	2.4 (±2.3)	5.4 (±2.2)	4.0 (±3.5)	4.0 (±2.6)	2.7 (±2.7)
Overall	2.7 (±2.4)		4.7 (±3.0)		3.4 (±2.7)	

SDs, standard deviations. Values in μm.

**Table 6:** Average scanning time (±standard deviation) for each scanning strategy.

	Strategy A		Strategy B		Strategy C	
	**Examiner 1**	**Examiner 2**	**Examiner 1**	**Examiner 2**	**Examiner 1**	**Examiner 2**
Session 1	93 (±16)	53 (±5)	84 (±14)	51 (±5)	80 (±12)	49 (±6)
Session 2	48 (±7)	52 (±6)	47 (±6)	47 (±4)	42 (±8)	46 (±6)
Overall	62 (±21)		57 (±18)		54 (±17)	

Values in seconds.

## Data Availability

Please contact the corresponding author for data requests.
